# Biocontrol of the Major Plant Pathogen *Ralstonia solanacearum* in Irrigation Water and Host Plants by Novel Waterborne Lytic Bacteriophages

**DOI:** 10.3389/fmicb.2019.02813

**Published:** 2019-12-06

**Authors:** Belén Álvarez, María M. López, Elena G. Biosca

**Affiliations:** ^1^Departamento de Bacteriología, Instituto Valenciano de Investigaciones Agrarias, Valencia, Spain; ^2^Departamento de Microbiología y Ecología, Universitat de València, Valencia, Spain

**Keywords:** bacterial wilt, environmental water, susceptible host, phage treatment, biological method, sustainable agriculture

## Abstract

Three new lytic bacteriophages were found to effectively control the pathogen *Ralstonia solanacearum*, a quarantine bacterium in many countries, and causative agent of bacterial wilt, one of the most important vascular plant diseases. Bacterial wilt management has been carried out with fluctuating effects, suggesting the need to find alternative treatments. In this work, three lytic phages were isolated from environmental water from geographically distant regions in Spain. They proved to specifically infect a collection of *R. solanacearum* strains, and some of the closely related pathogenic species *Ralstonia pseudosolanacearum*, without affecting non-target environmental bacteria, and were able to lyze the pathogen populations within a wide range of conditions comprising environmental values of water temperatures, pH, salinity, and lack of aeration found in storage tanks. The three bacteriophages displayed high efficiency in controlling *R. solanacearum*, with reductions of the bacterial populations of several orders of magnitude in just a few hours, and proved to be able to survive in freshwater for months at environmental temperatures keeping activity on *R. solanacearum*, pointing out their suitability for field application through irrigation. Concerning their biocontrol potential, they were effective in reducing high populations of the pathogen in environmental water, and bacterial wilt incidence *in planta* by watering with either one phage or their combinations in assays with more than 300 plants. This is the first report on effective *R. solanacearum* biocontrol by applying single or combined bacteriophages through irrigation water in conditions mimicking those of the natural settings. The three phages belong to the *Podoviridae* family and are members of the *T7likevirus* genus. They are the first isolated phages from river water with activity against *R. solanacearum*, showing the longest persistence in natural water reported until now for phages with biocontrol potential, and consistently being able to control the disease in the host plant under environmental conditions. Consequently, the use of these bacteriophages for the prevention and/or biocontrol of the bacterial wilt disease caused by *R. solanacearum* has been patented. Evidence provided reveals the suitability of these waterborne phages to be effectively considered as a valuable strategy within the frame of sustainable integrated management programs.

## Introduction

The highly pathogenic *Ralstonia solanacearum* bacterial species (Safni et al., 2014) has long belonged to the so-called *R. solanacearum* species complex, formed by heterogeneous strains classified into four phylotypes (Fegan and Prior, 2005), all of them causative agents of bacterial wilt (Kelman, 1953; Hayward, 1991). The complex was divided into three species, *R. solanacearum*, *Ralstonia pseudosolanacearum*, and *Ralstonia syzygii* subsp. *indonesiensis* (Safni et al., 2014; Prior et al., 2016), which are able to infect over 400 plant species worldwide, being a major threat to agriculture (Hayward, 1991; Elphinstone, 2005; Mansfield et al., 2012; EFSA Panel on Plant Health, 2019). These pathogens are soil and water borne, penetrate the host through the roots, and cause wilting by massively colonizing the xylem vessels and producing vascular dysfunction (Vasse et al., 1995; Álvarez et al., 2008b, 2010). The damage they cause has been related to the unusually high number of virulence and pathogenicity factors they synthesize (Schell, 2000; Poueymiro and Genin, 2009; Genin and Denny, 2012; Peeters et al., 2013; Deslandes and Genin, 2014).

The present *R. solanacearum* species is composed of strains of former phylotype II (Fegan and Prior, 2005), producing bacterial wilt in strategic crops for human supply, and also in ornamentals of economic importance (Elphinstone, 2005; Safni et al., 2014). It is considered a quarantine bacterium and a pest of economic and environmental importance in the European Union (EU) (Anonymous, 2000; EFSA Panel on Plant Health, 2019) and a Select Agent in the United States (Lambert, 2002). Solanaceous plant species are major hosts of this pathogen all around the world (Elphinstone, 2005). A major concern is the establishment of *R. solanacearum* in the environment. Thus, occurrence of outbreaks has been linked to the presence of this pathogen in environmental reservoirs, mainly water (Elphinstone et al., 1998; van Elsas et al., 2000, 2001; Caruso et al., 2005, 2017), where it can survive for years as a free-living form and/or in roots of semiaquatic weeds or other reservoir plants, retaining pathogenicity (Álvarez et al., 2008a, 2010; Hong et al., 2008). This poses a problem to growers, especially in areas where water is a scarce commodity, and particularly when there is a ban on irrigation of host plants with *R. solanacearum* contaminated water, as in the EU countries (Anonymous, 1998, 2006; EFSA Panel on Plant Health, 2019).

No management method seems to be fully advisable against bacterial wilt, since crop protection chemicals do not provide enough control and usually have a harmful impact on the environment and/or the human health, favor the emergence of resistances and are expensive, physical treatments are quite ineffective, crop rotations are often impractical, and the pathogen displays high aggressiveness and endurance in adverse environmental conditions (López and Biosca, 2005; Álvarez et al., 2010; Yuliar et al., 2015; Nicolopoulou-Stamati et al., 2016; Cao et al., 2018). Bacterial wilt biocontrol strategies have also been proposed mainly based on the antagonistic effect of other bacterial species, genetically modified strains of *R. solanacearum* and/or the activity of bacteriophages (Yamada, 2012; Hanemian et al., 2013; Yuliar et al., 2015; Álvarez and Biosca, 2017; Cao et al., 2018; Wang et al., 2019), with quite different results. Among them, phage therapy with lytic phages is being regarded as one of the most promising to provide valuable alternative for controlling pathogenic bacteria (Abedon et al., 2017; Svircev et al., 2018). Lytic phages can effectively and specifically lyze their bacterial target without impact on the surrounding microbiota. They are self-replicating over the course of treatment and self-limiting once the target has been destroyed, and considered safe natural products with relatively low production costs (Loc-Carrillo and Abedon, 2011; Abedon et al., 2017), easy to integrate in a sustainable agricultural system, with less legal restrictions than chemicals. In fact, phages have already been proposed for biocontrol of important plant diseases (Jones et al., 2007; Balogh et al., 2010; Doffkay et al., 2015; Buttimer et al., 2017; Svircev et al., 2018).

Bacteriophage-based bacterial wilt biocontrol has been described with either lytic or lysogenic bacteriophages (Tanaka et al., 1990; Yamada et al., 2007; Fujiwara et al., 2011; Addy et al., 2012a, b; Bae et al., 2012; Kalpage and De Costa, 2014; Bhunchoth et al., 2015; Wei et al., 2017), which were proved to be active against strains belonging to *R. pseudosolanacearum* and/or *R. syzygii* subsp. *indonesiensis* but, not to the present *R. solanacearum*, and only two of them demonstrated potential for biocontrol *in planta* (Álvarez and Biosca, 2017). In this work, a complete screening of specificity, stability, and lytic activity of a selection of new *R. solanacearum* phages isolated from environmental water was performed under diverse environmental conditions, which allowed for an assessment of their biocontrol activity in both irrigation water and host plants with different phage combinations, and the development of an innovative environmentally friendly method for a safe and sustainable agriculture, which will be easily and effectively applied in the field through irrigation water.

## Materials and Methods

### Bacterial Strains and Growth Conditions

*Ralstonia solanacearum* strain IVIA-1602.1 (also CFBP 4944 or DSMZ 100387) isolated from potato (*Solanum tuberosum*) in

Spain was routinely used as the bacterial host. For specificity assays, a collection of 35 *R. solanacearum* strains (Table 1), as well as seven *R. pseudosolanacearum* strains (Table 1), and 13 strains of other phytopathogenic bacterial species (Table 2) were used. All strains were kept at −80°C in a 30% (vol/vol) glycerol medium. *R. solanacearum* and *R. pseudosolanacearum* strains were grown on the non-selective Yeast Extract Peptone Glucose Agar (YPGA) (Lelliot and Stead, 1987) for 48 h at 28°C. *Clavibacter michiganensis* subsp. *michiganensis*, *Xanthomonas campestris* pv. *campestris*, and *Xanthomonas* sp. strains were grown on YPGA for 48 h at either 26 or 28°C, respectively. *Dickeya* sp., *Erwinia amylovora*, *Pectobacterium atrosepticum*, *Pectobacterium carotovorum*, and *Pseudomonas savastanoi* pv. *savastanoi* strains were grown on King’s medium B (KB) (King et al., 1954) for 48 h at 25°C. *Rhizobium radiobacter* and *Rhizobium rhizogenes* strains were grown on general Mannitol Glutamate Luria (MGL) medium (Angelosi et al., 1991) for 48 h at 28°C. For initial environmental impact assays, a collection of 46 unidentified bacterial isolates from river water (RW), and from tomato (*Solanum lycopersicum*) soil, rizhosphere and plant endophytes were grown either on YPGA, KB, Luria Bertani (LB) (Miller, 1987), or Wilbrink (WB) (Koike, 1965) general media for 48 h at 28°C.

**TABLE 1:** *Ralstonia solanacearum* and *R. pseudosolanacearum* strains tested for bacteriophage specificity in this work.

**Strain code**	**Bv^∗^**	**Country of origin**	**Host**	**Activity against vRsoP-**		
				**WF2**	**WM2**	**WR2**
***Ralstonia solanacearum***						
NCPPB^†^1115	2	United Kingdom	*Solanum tuberosum*	**+**	**+**	**+**
NCPPB 1584	2	Cyprus	*S. tuberosum*	+	+	+
NCPPB 2505	2	Sweden	*S. tuberosum*	+	+	+
NCPPB 2797	2	Sweden	*S. dulcamara*	+	+	+
BR 264	2	United Kingdom	*S. dulcamara*	+	+	+
Bordeaux 11-47	2	France	*S. melongena*	+	+	+
Nantes 9-46	2	France	*S. lycopersicum*	+	+	+
550	2	Belgium	*S. tuberosum*	+	+	+
IPO-1609	2	The Netherlands	*S. tuberosum*	+	+	+
Port 448	2	Portugal	*S. tuberosum*	+	+	+
W 12	2	Belgium	*S. tuberosum*	+	+	+
WE 4-96	2	United Kingdom	River water	+	+	+
Tom 1	2	United Kingdom	*S. lycopersicum*	+	+	+
IVIA^§^-1602.1	2	Spain (Canary Islands)	*S. tuberosum*	+	+	+
IVIA-2049.53	2	Spain (Canary Islands)	Soil	+	+	+
IVIA-2068.58a	2	Spain (Canary Islands)	*S. tuberosum*	+	+	+
IVIA-2068.61a	2	Spain (Canary Islands)	*S. tuberosum*	+	+	+
IVIA-2093.3.1	2	Spain (Canary Islands)	*S. tuberosum*	+	+	+
IVIA-2093.5T.1a	2	Spain (Canary Islands)	*S. tuberosum*	+	+	+
IVIA-2128.1b	2	Spain (Castile and León)	*S. tuberosum*	+	+	+
IVIA-2128.3a	2	Spain (Castile and León)	*S. tuberosum*	+	+	+
IVIA-2167.1a	2	Spain (Castile and León)	River water	+	+	+
IVIA-2167.2b	2	Spain (Castile and León)	River water	+	+	+
IVIA-2528.A_1__–__2_	2	Spain (Castile and León)	River water	+	+	+
IVIA-2528.A_3_._1_	2	Spain (Castile and León)	River water	+	+	+
IVIA-2528.54.A_2_	2	Spain (Castile and León)	River water	+	+	+
IVIA-2751.11	2	Spain (Estremadura)	River water	+	+	+
IVIA-2762.1	2	Spain (Estremadura)	*S. lycopersicum*	+	+	+
IVIA-2762.4	2	Spain (Estremadura)	*S. lycopersicum*	+	+	+
IVIA-3090.1	2	Spain (Andalusia)	*S. lycopersicum*	+	+	+
IVIA-3090.5	2	Spain (Andalusia)	*S. lycopersicum*	+	+	+
IVIA-3205.A.22	2	Spain (Castile-La Mancha)	River water	+	+	+
IVIA-3243	2	Spain (Andalusia)	*S. lycopersicum*	+	+	+
IVIA-3359.9	2	Spain (Castile-La Mancha)	River water	+	+	+
IVIA-3359.10	2	Spain (Castile-La Mancha)	River water	+	+	+
***R. pseudosolanacearum***						
GMI-1000	1	French Guiana	*S. lycopersicum*	+	+	+
NCPPB 325	1	USA	*S. lycopersicum*	−	−	−
NCPPB 3996	3	Peru	*S. lycopersicum*	+	+	+
NCPPB 3997	3	Australia	*S. tuberosum*	−	−	−
NCPPB 4029	4	Sri Lanka	*S. tuberosum*	+	+	+
NCPPB 4005	4	Philippines	*Zingiber* sp.	−	−	−
NCPPB 4011	5	China	*Morus* sp.	−	−	−

*^∗^Bv, biovar, a classification of strains based on biochemical properties (Hayward, 1991). The former *R. solanacearum* species complex comprised five biovars. ^†^NCPPB, National Collection of Plant Pathogenic Bacteria, United Kingdom. ^§^IVIA, Collection of Plant Pathogenic Bacteria, Valencian Institute for Agricultural Research, Spain.*

**TABLE 2:** Phytopathogenic bacterial species and strains tested for bacteriophage specificity in this work.

**Bacterial species**	**Strain code**	**Country of origin**	**Host**
*Clavibacter michiganensis* subsp. *michiganensis*	IVIA^§^-873	Spain	*Solanum lycopersicum*
*Dickeya* sp.	IVIA-4830	Spain	*S. lycopersicum*
*Erwinia amylovora*	CFBP^†^-1430	France	*Crataegus oxyacantha*
*E. amylovora*	IVIA-1892.1	Spain	*Pyrus communis*
*Pectobacterium atrosepticum*	IVIA-3447	Spain	*S. tuberosum*
*P. carotovorum*	IVIA-3902	Spain	*S. lycopersicum*
*Pseudomonas savastanoi* pv. *savastanoi*	IVIA-1628-3	Spain	*Olea europaea*
*P. savastanoi* pv. *savastanoi*	IVIA-1657-8	Spain	*O. europaea*
*Rhizobium radiobacter*	B6	USA	*S lycopersicum*
*R. radiobacter*	C58	USA	*Prunus avium*
*R. rhizogenes*	K84	Australia	Nonpathogenic
*Xanthomonas campestris* pv. *campestris*	IVIA-1147	Spain	*Brassica oleracea*
*Xanthomonas* sp.	IVIA-3617	Spain	*Capsicum annuum*

*^§^IVIA, Collection of Plant Pathogenic Bacteria, Valencian Institute for Agricultural Research, Spain. ^†^CFBP, French Collection of Phytopathogenic Bacteria, France.*

### Detection and Isolation of Bacteriophages From Environmental Water

A collection of *R. solanacearum* lytic bacteriophages was isolated according to European Directives (Anonymous, 1998, 2006) from several rivers in different geographically distant regions of Spain, in the vicinity of potato and tomato fields formerly affected by bacterial wilt, and subsequently eradicated (Caruso et al., 2017). Bacteriophages were isolated by enrichment detection assays for *R. solanacearum* lytic phages according to Álvarez et al. (2007), with minor modification. Briefly, 1-mL aliquots of 0.22-μm-filtered RW samples were added to 5 mL of growing cells of *R. solanacearum* suspensions of strain IVIA-1602.1 [O.D. at 600 nm = 1, about 10^9^ colony-forming units (CFU/mL)] in a modified Wilbrink broth (MWB) (Caruso et al., 2002). Bacterial suspensions without filtered water were used as negative controls. Incubations were done overnight with shaking (200 rpm) at 28°C. The lysates obtained were 10-fold serial diluted in 10 mM phosphate buffered saline (PBS) buffer at pH 7.2 and plated onto YPGA previously inoculated with *R. solanacearum* strain IVIA-1602.1 according to a standard surface plating method (Civerolo, 1990). A selection of plaques from the collection of the bacteriophages was purified and their lytic activity verified against *R. solanacearum* liquid cultures in MWB.

### Specificity and Physiological Characteristics of the Bacteriophages

The specificity against the *R. solanacearum* species was determined for each of the bacteriophages tested by standard method in both, liquid and solid media, with 35 *R. solanacearum* and seven *R. pseudosolanacearum* strains from different hosts and geographical origins (Table 1), and 13 strains of other phytopathogenic bacterial species (Table 2). Moreover, 46 unidentified environmental bacterial isolates were also tested, including 23 from different RW samples, eight from tomato field soil, eight from tomato rhizosphere, and seven tomato endophytes. Briefly, 1-mL or 10–20 μL of 0.22-μm-filtered lysates were added to 5 mL of bacterial suspensions (O.D. at 600 nm = 1) or dropped onto bacterial lawns, respectively, for liquid or solid general media, depending on the bacterial species. Incubation conditions for lytic activity were those required for growth of each bacterial species. All assays were performed at least in duplicate.

Physiological characteristics of the bacteriophages tested were evaluated by lytic activity assays against *R. solanacearum* strain IVIA-1602.1 as abovementioned. Lytic activity was determined in MWB at temperature values from 4 to 39°C, and environmental water at temperature and pH values within environmental range from 14 to 29°C and 6.5 to 9.0, respectively. The effect of salinity on lytic activity was tested in water samples of different origins (river, lake, and irrigation) and saline concentrations around 1.5–2.0 and 35%. Lytic activity was also determined in both, aeration by shaking at 200 rpm and static conditions, in MWB at 28°C. In all conditions, incubation was carried out until observation of lytic activity. All assays were performed at least in duplicate.

### Morphology of the Bacteriophages

Viral particles of selected bacteriophages were visualized by transmission electron microscopy (TEM) after negative staining with a solution of 1% phosphotungstic acid (PTA) in water, adjusted to pH 7 with 1 N NaOH solution. Drops of 0.22-μm-filtered lysates were placed on formvar and carbon covered grids. After 30–60 s drying, the excess liquid was blotted off, and drops of PTA solution were added on top of the lysate drops. After 1 min drying, the excess liquid was blotted off. Grids were air dried before TEM examination.

### Kinetics of the Lytic Activities of the Bacteriophages

The growth dynamics between *R. solanacearum* strain IVIA-1602.1 and each of the bacteriophages tested was initially performed in optimal conditions by coinoculation of the strain at about 10^9^ CFU/mL and the phage tested at about 10^9^ plaque-forming units (PFU)/mL, multiplicity of infection (MOI) = 1, both incubated in MWB at 28°C with shaking (200 rpm). The dynamics was subsequently performed between *R. solanacearum* and one selected bacteriophage by coinoculation of the strain at about 10^6^ CFU/mL and the selected phage at about 10^4^–10^3^ PFU/mL, both incubated in MWB at 24 and 14°C with shaking (200 rpm). Additionally, the kinetics between the strain and the selected bacteriophage was monitored by using a Bioscreen C apparatus (Labsystems) with a discontinuous shaking regimen (several sec each 10 min). Thus, strain IVIA-1602.1 and the phage were coinoculated at different proportions in MWB, with the strain at about 10^9^ CFU/mL and the phage from 10^9^ to 10^1^ PFU/mL, and incubated at 26°C. The O.D._580 nm_ of the liquid cocultures were determined every 30 min and reported as the mean of five different measurements.

### Survival of the Bacteriophages in Environmental Water

River water from two regions in Spain was used to test survival of selected bacteriophages. One of the regions was a *R. solanacearum*-free area, Southeastern (SE) region, with environmental water displaying temperature values from 11.5 to 22.0°C and mean pH value around 8.13, and the other region an originally *R. solanacearum*-contaminated area, Northwestern (NW) region, with environmental water displaying temperature values from 3.5 to 20.9°C and mean pH value around 7.36. With respect to the composition of the RW samples, SE-RW had values about 100 times higher for Mn, 10 times higher for Fe, between 5 and 10 times higher for chlorides and triple nitrates than NW-RW and mean pH value of 8.1, whereas NW-RW had values about four times higher for phosphate than SE-RW and mean pH value of 7.2. Both types of water were 0.22-μm-filtered and autoclaved previously to the survival experiments.

For each of the SE-RW and NW-RW samples, three microcosms were prepared, each of them with a 300-mL volume of water, which were separately inoculated with the 0.22-μm-filtered lysates of the bacteriophages tested at a final concentration ranging from 10^7^ to 10^5^ PFU/mL. The microcosms were incubated at 4, 14, and 24 ± 1°C, and sampled for plaque counts at inoculation time (day 0), 1, 2, 3, 5 weeks postinoculation (wpi), 3, 5 months (mpi), and 3 years (ypi), performed from each microcosm according to a standard surface plating method after 48 h at 28°C. Survival experiments were performed in two separate assays at least in duplicate.

### Bacteriophage-Based *R. solanacearum* Biocontrol Activity in Environmental Water and Host Plants

#### Biocontrol in Environmental Water

##### In the absence of water microbiota

Volumes of 100 mL of 0.22-μm-filtered and autoclaved RW samples were inoculated with *R. solanacearum* strain IVIA-1602.1 at about 5 × 10^6^ CFU/mL and each of the bacteriophages tested at about 10^4^ PFU/mL (MOI = 0.01). Incubation was done with shaking (200 rpm) over 30 h at 24°C and 60 h at 14°C. Aliquots for bacterial and phage counts were regularly taken, serially 10-fold diluted in PBS and plated onto YPGA as abovementioned. Assays were done at least in duplicate, in separate experiments.

##### In the presence of water microbiota

River water samples with the whole microbiota (protozoa, bacteria, virus) were processed by enrichment assays for detection of *R. solanacearum* lytic phages that could be naturally present in the water samples. As the assays did not detect any initial lytic activity, the presence of *R. solanacearum* indigenous phages was discarded, and the water samples were then inoculated with a selected bacteriophage at about 10^4^ PFU/mL. Volumes of 100 mL were processed in three different ways as follows: (i) 0.22-μm-filtration and autoclaving, with no alive microbiota (AW), (ii) 0.22-μm-filtration, only containing the indigenous phages without activity against *R. solanacearum* and the inoculated phage (FW), or (iii) without treatment, with the whole native microbiota and the inoculated phage (W). All of them were subsequently inoculated with *R. solanacearum* strain IVIA-1602.1 at about 5 × 10^6^ CFU/mL (MOI = 0.01). Incubation was done in static conditions at 24 and 14°C for a month. Aliquots for *R. solanacearum* counts were taken at inoculation time (day 0), at 30 h at 24°C and 60 h at 14°C, and then weekly for a month, serially 10-fold diluted in PBS and plated onto YPGA as abovementioned. Assays were done at least in duplicate, in separate experiments.

#### Biocontrol in Host Plants by Watering

##### With one single phage

The ability of a selected RW bacteriophage for bacterial wilt biocontrol *in planta* was tested in a susceptible host. Sets of 10 tomato plants cv. Roma aged 3 weeks were coinoculated in duplicate in two independent experiments (Experiment 1 and Experiment 2) by watering once with 20 mL of *R. solanacearum* strain IVIA-1602.1 at concentrations of 10^4^ or 10^5^ CFU/mL, and a selected bacteriophage at concentrations of 10^9^ or 10^6^ PFU/mL and their 10-fold dilutions. Another experiment (Experiment 3) was performed in triplicate by watering once with 20 mL of *R. solanacearum* strain IVIA-1602.1 at 10^5^ CFU/mL and the same selected bacteriophage at 10^8^ PFU/mL. Tomato plants inoculated either with the bacterial strain or non-sterile water were also included as controls. Plants were maintained in a climatic chamber of adequate dimensions, in cycles of 16 h light 8 h darkness at 26°C and 70% humidity in optimal conditions for disease development, under Biosecurity Level 3 (BSL3). After inoculation, plants were monitored periodically for symptom development by visual inspection during 6–8 weeks. Wilting symptoms in less than 25% of leaves were recorded as first symptoms to be confirmed in subsequent evaluations. Progressive increase in wilting incidence to 50–75% of the leaves and branches was recorded as positive wilting in the plant. More than 75% of leaves and branches affected were recorded as near collapse or collapse of the wilted plant. Bacterial reisolation from wilted plants was performed according to European Directives (Anonymous, 1998, 2006) in a modified semiselective medium South Africa (SMSA) agar (Elphinstone et al., 1996) followed by PCR identification of the colonies (Anonymous, 2006).

##### With single phages and their combinations

The abilities of selected RW phages for bacterial wilt biocontrol *in planta*, either alone or taking part of a phage mixture, were tested in a susceptible host in biocontrol assays. Sets of around 35 tomato plants cv. Roma aged 4–5 weeks were coinoculated in two independent experiments by watering once with 20 mL of *R. solanacearum* strain IVIA-1602.1 at 10^5^ or 10^6^ CFU/mL and the selected bacteriophages and their combinations at 10^7^ PFU/mL (MOIs = 100 and 10, respectively) in all the experimental conditions. Tomato plants inoculated either with the bacterial strain or non-sterile water were also included as controls. Plants (more than 300 per assay) were incubated under BSL3 conditions, monitored for a period of 1.5–2 months, and then sampled and processed as abovementioned.

##### Statistical analysis

For each biocontrol assay in host plants, the relation among different treatments (bacterium, bacterium and phage in every combination, water) and wilting *versus* no wilting were compared using the Chi-square test. A *p*-value below 0.05 was considered significant. Data were analyzed with SPSS 20.0 software (IBM SPSS Statistics).

### Sequencing of the Bacteriophage Genomes and Initial Analysis

Molecular biology techniques for DNA isolation and sequencing were performed (Sambrook and Russell, 2001; Pickard, 2009). Genomic DNA was isolated from each of the selected bacteriophages. Briefly, concentrated phage suspensions were obtained from the 0.22-μm-filtered lysates, which were treated with DNAse and RNAse at the same final concentration of 1 μg/mL, and polyethylene glycol 8000 1:10 (w/v) for precipitation of viral particles. After treatment with SDS 20% and protease K at 20 mg/mL to lyze the particles, the genomic DNA was obtained from the upper phase after mixture with phenol, chloroform, and isoamyl alcohol at 25:24:1 (v/v), and precipitated by the addition of ethanol, progressively at 100 and 70%. The DNA pellets were dried and resuspended in nuclease-free water prior to checking DNA concentrations and purities by UV spectrophotometry.

Then, massive-scale sequencing was carried out by Illumina SBS Technology. Paired-end libraries with approximate average insert lengths of 200 base pairs were synthesized from the DNA using the NexteraXT (Illumina) protocol, and library concentrations and sizes were assayed on a 2100 Bioanalyzer. Libraries were sequenced using an Illumina sequencer HiSeq 2500/1500 (2 × 150 bp). The obtained sequences were analyzed and preliminary annotated after cleaning of the sequences of the FASTQ file. Low-quality sequences (reads with a base quality less than 20) were removed, and only the high-quality reads were assembled to construct unique consensus sequences by analysis against a reference sequence and *de novo* genome assembly. For each bacteriophage genome, a single large contig was generated by reads assembly, and genomes were defined as complete if the single contig assembled without error. These contigs were used to identify closely related homologs with the NCBI BlastN tool by comparison with the sequences published in the GenBank. Preliminary functional annotations of the assembled genomes were also performed by sequence comparison with the NCBI public database, and the best aligning results were selected to annotate the unigenes.

## Results

### Three Bacteriophages Isolated From Environmental Water Lyze *R. solanacearum*

**FIGURE 1:**
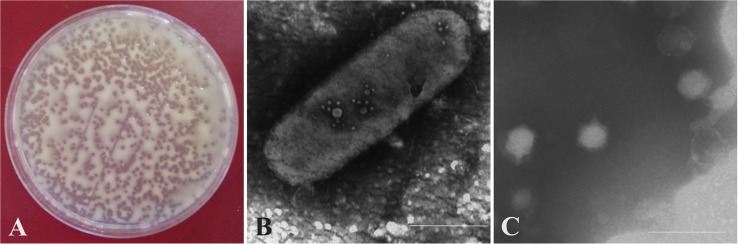
Morphology of the *Ralstonia solanacearum*-infecting bacteriophages. Representative pictures for one of the three phages are shown. **(A)** Plaques of vRsoP-WF2 on a lawn of *R. solanacearum* strain IVIA-1602.1 on Yeast Peptone Glucose Agar. Viral particles of vRsoP-WF2 visualized by transmission electron microscopy, either **(B)** attached on the cell surface of strain IVIA-1602.1 or **(C)** displaying typical polygonal heads and short tails of the *Podoviridae*.

An initial collection of lytic bacteriophages was isolated from former *R. solanacearum* contaminated areas in Spain. They were purified, and eight of them selected according to their plaque size and different geographical origins. These were further characterized in a range of environmental conditions, and then three of them selected on the basis of their lytic characteristics, mainly specificity against *R. solanacearum*, kinetics of their lytic activity, and survival in environmental water. They were isolated in different years from RW from geographically distant Spanish regions that do not share any tributary with each other. Bacteriophages were initially named F2 (isolated from Castile and León, 2001), M2 (from Estremadura, 2003), and R2 (from Andalusia, 2004). They produced transparent plaques on a lawn of *R. solanacearum* after 36–48 h on YPGA. Figure 1A shows a representative picture of F2 plaques on a lawn of strain IVIA-1602.1 on YPGA.

Lytic activity of the three phages was positive for 35 strains of *R. solanacearum* (former phylotype II) from different sources, hosts and years of isolation, all of them of biovar 2 (Table 1). Among them, 13 were from different countries and/or reference strains and the remaining strains were isolated in Spain. Lytic activity was variable against seven strains of *R. pseudosolanacearum* (former phylotypes I and III) of biovars 1, 3, 4, and 5 (Table 1). Lytic activity was negative against 13 strains of phytopathogenic bacterial species from other genera (Table 2) and 46 unidentified environmental bacterial isolates from different RW samples, and tomato soil, rhizosphere, and endophytes. The main genera tested were *Acinetobacter*, *Aeromonas*, *Alcaligenes*, *Moraxella*, and *Pseudomonas* for water isolates; *Arthrobacter*, *Bacillus*, *Pseudomonas*, *Sphingobacterium*, and *Streptomyces* for soil isolates; *Arthrobacter*, *Pantoea*, *Pseudomonas*, *Serratia*, and *Streptomyces* for rhizosphere isolates; and *Bacillus*, *Enterobacter*, and *Sphingomonas* for endophytes. These results demonstrate the specificity of the phages for *R. solanacearum* and also for some strains of the closely related species *R. pseudosolanacearum*. Further, lytic activity of the three phages against the strain IVIA-1602.1 of *R. solanacearum* was positive between 14 and 31°C (positive at 14, 20, 24, 28, and 31°C), and negative at 9 and between 32 and 39°C (negative at 4, 9, 32, 35, 37, and 39°C), in MWB at pH 7. In water from river, lake, and irrigation sources, phage activity was positive at pH values ranging between 6.5 and 9.0 at 14, 20, and 29°C. Lytic activity was also positive in freshwater from different sources with salinity contents up until approximately 2.1% (positive at 1.58% pH 7.7, pH 8; 2.1% pH 7.8), and negative in saline water at 35% pH 8 from seaport and beach. With respect to aeration, lytic activity was similarly observed after incubation in MWB at 28°C with aeration by shaking at 200 rpm as well as in static conditions, being faster with aeration.

### The Three Bacteriophages F2, M2, and R2 Belong to the *Podoviridae* Family

Viral particles adsorbed to the host cell and viral shapes were observed in detail by TEM of the bacteriophage lysates. Figure 1B shows a representative picture of F2 viral particles on the cell surface of strain IVIA-1602.1. The three phages displayed polygonal heads of 40–60 nm in diameter and short tails (Figure 1C), a morphology characteristic of bacteriophages from the *Podoviridae* family. Bacteriophages F2, M2, and R2 were then renamed vRsoP-WF2, vRsoP-WM2, and vRsoP-WR2, respectively, where “v” stands for “virus,” “Rso” for *R. solanacearum*, “P” for “podovirus,” and “W” for “water.”

### vRsoP-WF2, vRsoP-WM2, and vRsoP-WR2 Effectively Lyze *R. solanacearum* Cells

**FIGURE 2:**
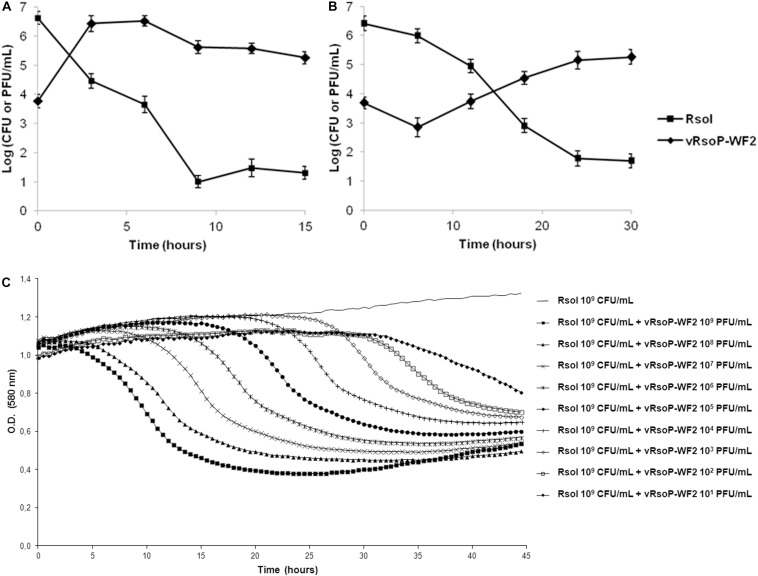
Time course of the interaction between *Ralstonia solanacearum* and the bacteriophages. Representative data for one of the three phages are shown. Cocultures of strain IVIA-1602.1 at 10^6^ colony-forming units (CFU)/mL and the phage vRsoP-WF2 at 10^4^–10^3^ plaque-forming units (PFU)/mL in modified Wilbrink Broth (MWB) monitored at **(A)** 24 and **(B)** 14°C. Culturable bacterial cells and plaque counts of the phage were performed on Yeast Peptone Glucose Agar. Points are the means for two separate assays in duplicate, and error bars indicate variation as the standard deviation for each point. **(C)** Cocultures of strain IVIA-1602.1 at 10^9^ CFU/mL and the phage vRsoP-WF2 at 10^9^ PFU/mL and their 10-fold dilutions to 10^1^ PFU/mL in MWB monitored at 26°C. Reductions in bacterial populations were visualized as decreases in optical density (O.D.) values at 580 nm. Points are the means for five replicates, and standard deviation intervals range ± 0.01 O.D. (580 nm).

Reductions in the inoculated bacterial populations caused by the lytic action of the phages were observed when monitoring cocultures of strain IVIA-1602.1 at 10^9^ CFU/mL and each of the three phages at 10^9^ PFU/mL in MWB at 28°C, since initial turbid suspensions became transparent in 5–6 h, corresponding to bacterial concentrations of 10^4^–10^3^ CFU/mL, for each of the bacteriophages tested. Reductions were also observed and quantified in cocultures of strain IVIA-1602.1 at 10^6^ CFU/mL and the phage vRsoP-WF2 at 10^4^–10^3^ PFU/mL in MWB at 24 and 14°C, since the inoculated bacterial populations decreased 5–6 log units in 9 h postinoculation (hpi) at 24°C (Figure 2A), and 4–5 log units in 24 hpi at 14°C (Figure 2B), so being faster and more effective at 24°C. The dynamics was more carefully monitored for cocultures of strain IVIA-1602.1 at 10^9^ CFU/mL and the phage vRsoP-WF2 at 10^9^ PFU/mL and their 10-fold dilutions to 10^1^ PFU/mL in MWB at 26°C (Figure 2C). Reductions in the inoculated bacterial populations were observed in all cases, correlated to the phage concentration, so that the less phage is added the more time is needed to reduce the population of bacterial cells, with the proportion strain IVIA 1602.1 (10^9^ CFU/mL): vRsoP-WF2 (10^9^ PFU/mL) being the most effective, since the reduction achieved in *R. solanacearum* populations was the highest in less than 24 hpi.

### vRsoP-WF2, vRsoP-WM2, and vRsoP-WR2 Displayed Long-Term Survival in the Absence of *R. solanacearum*, Keeping Lytic Activity

**FIGURE 3:**
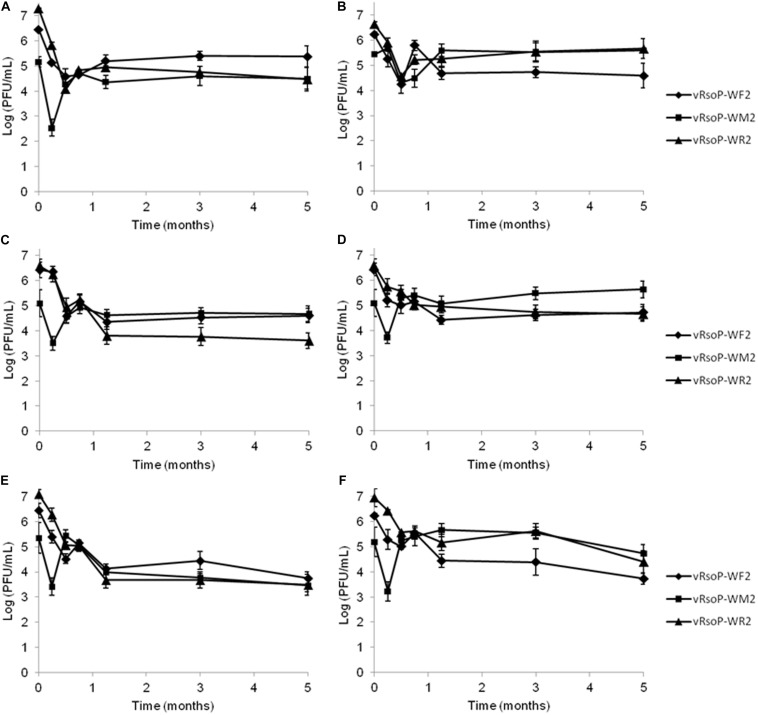
Survival of the *Ralstonia solanacearum*-infecting bacteriophages in environmental water. Population sizes of vRsoP-WF2, vRsoP-WM2, and vRsoP-WR2 referred to plaque-forming units (PFU)/mL detected with lytic activity over 5 months in **(A,C,E)** Northwestern river water (NW-RW) and **(B,D,F)** Southeastern river water (SE-RW) microcosms in the absence of *R. solanacearum* at **(A,B)** 4, **(C,D)** 14, and **(E,F)** 24°C. Plaque counts of the phages were performed on Yeast Peptone Glucose Agar. Points are the means for two separate assays in duplicate, and error bars indicate variation as the standard deviation for each point.

All survival curves for the three phages are shown in Figure 3. Upon inoculation of vRsoP-WF2, vRsoP-WM2, and vRsoP-WR2 in different environmental water samples (SE-RW and NW-RW), and incubation at 4, 14, and 24°C in the absence of any bacterial host, declines in phage populations were observed within the first mpi, followed by stabilization at the levels of 10^4^–10^5^ PFU/mL at 4 and 14°C, and 10^3^–10^4^ PFU/mL at 24°C until 5 mpi (Figure 3). The three bacteriophages maintained their lytic activity against the strain IVIA 1602.1 after more than 5 months of being incubated in two different environmental water microcosms, and up to 3 years at 4 and 14°C, although volumes of phage suspensions 10 times higher than usual were necessary to detect lytic activity, demonstrating their high stability in environmental water, in the absence of any bacterial host. Their lytic activity after 3 years, although positive, was less effective, since smaller plaques were obtained. Similar results were observed for SE-RW and NW-RW samples despite their different physical and chemical properties.

### Bacteriophage-Based *R. solanacearum* Biocontrol in Environmental Water

#### In the Absence of Water Microbiota

**FIGURE 4:**
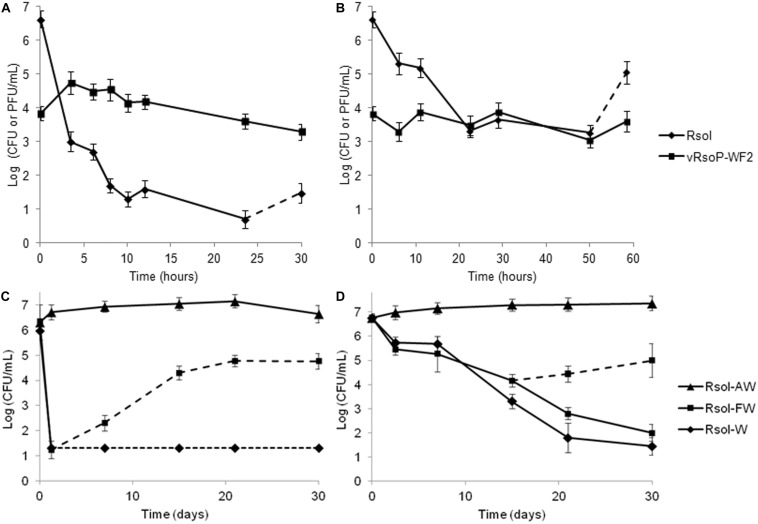
*Ralstonia solanacearum* biocontrol in environmental water by the bacteriophages. Representative data for one of the three phages are shown. In the absence of indigenous water microbiota. Cocultures of strain IVIA-1602.1 at 10^7^–10^6^ colony-forming units (CFU)/mL and the phage vRsoP-WF2 at 10^4^–10^3^ plaque-forming units (PFU)/mL in environmental water monitored at **(A)** 24 and **(B)** 14°C. Culturable bacterial cells and plaque counts of the phage were performed on Yeast Peptone Glucose Agar. In the presence of indigenous water microbiota. Strain IVIA-1602.1 was inoculated at 10^7^–10^6^ CFU/mL in: (AW) filtered and autoclaved, (FW) filtered, and (W) untreated environmental water previously inoculated with the phage vRsoP-WF2 at 10^4^–10^3^ PFU/mL and monitored at **(C)** 24 and **(D)** 14°C. **(A–D)** Dashed line represents the emergence of strain IVIA-1602.1 phage-resistant variants. **(C)** Dotted line represents unidentified bacterial colonies isolated from the environmental water since no *R. solanacearum* colonies were observed. Points are the means for two separate assays in duplicate, and error bars indicate variation as the standard deviation for each point.

Biocontrol activity by the phage vRsoP-WF2 against the strain IVIA-1602.1 occurred in sterile environmental water just inoculated with the phage and the bacterium, in the absence of water microbiota at both, 24 and 14°C. For initial phage and bacterial concentrations of about 10^4^ PFU/mL and 5 × 10^6^ CFU/mL, at 24°C bacterial populations decreased to values around 5 × 10^1^ CFU/mL (5 log units) within 10 hpi, with one additional log unit decrease by 24 hpi (Figure 4A), whereas at 14°C bacterial populations decreased to about 5 × 10^3^ CFU/mL (3 log units) within 23 hpi, keeping these levels in the following 24 hpi (Figure 4B). These data demonstrated the biocontrol ability of vRsoP-WF2 against *R. solanacearum* in sterile environmental water. Afterward, resistant bacterial cells appeared at both temperatures. These resistant variants were isolated and purified on YPGA, and PCR-identified as *R. solanacearum*. Lytic activity assays performed with these *R. solanacearum* variants and the phage vRsoP-WF2 confirmed the resistance of the isolated variants to the action of the lytic phage. On the other hand, at 24 and 14°C phage populations kept their levels, initially increasing only at 24°C and then stabilizing (Figures 4A,B).

#### In the Presence of Water Microbiota

To evaluate whether the appearance of *R. solanacearum* cells resistant to the action of the lytic phages could really take place in the environment, biocontrol assays were performed in non-sterile environmental water with the whole indigenous microbiota, where inoculated bacterial cells of strain IVIA-1602.1 were also exposed to the action of vRsoP-WF2 and monitored in the long term at 24 and 14°C. Results can be observed in Figures 4C,D. Inoculated bacterial populations maintained levels similar to the initial ones only in the control microcosms, containing filtered and autoclaved environmental water (AW) (Figures 4C,D), pointing out absence of biocontrol activity. Conversely, strong reductions in the inoculated bacterial populations were achieved in the rest of microcosms containing either filtered or untreated water (FW or W) (Figures 4C,D). However, although biocontrol activity occurred in both types of microcosms, effective long-term biocontrol was observed only in untreated water (W), in the presence of the whole native microbiota, under conditions mimicking those of natural settings. Phage-resistant bacterial cells appeared in the microcosms with filtered water (FW), only containing the inoculated bacteria facing vRsoP-WF2, at both temperatures (Figures 4C,D). In fact, at 14°C simultaneity of bacterial types, inoculated wild *R. solanacearum* strain IVIA-1602.1 and phage-resistant variants, were grown on plates. These variants were isolated and purified on YPGA, and PCR-identified as *R. solanacearum*. Lytic activity assays with these *R. solanacearum* variants and the phage vRsoP-WF2 confirmed the resistance of the bacterial isolates to the phage. In the long term, subsequent bacterial increases due to these resistant variants were observed only in filtered water (FW) (Figures 4C,D). No resistant cells were detected in untreated water (W) (Figures 4C,D). These data demonstrated the biocontrol ability of vRsoP-WF2 against *R. solanacearum* in non-sterile environmental water.

### Bacteriophage-Based *R. solanacearum* Biocontrol in Host Plants by Watering

#### With One Single Phage

**FIGURE 5:**
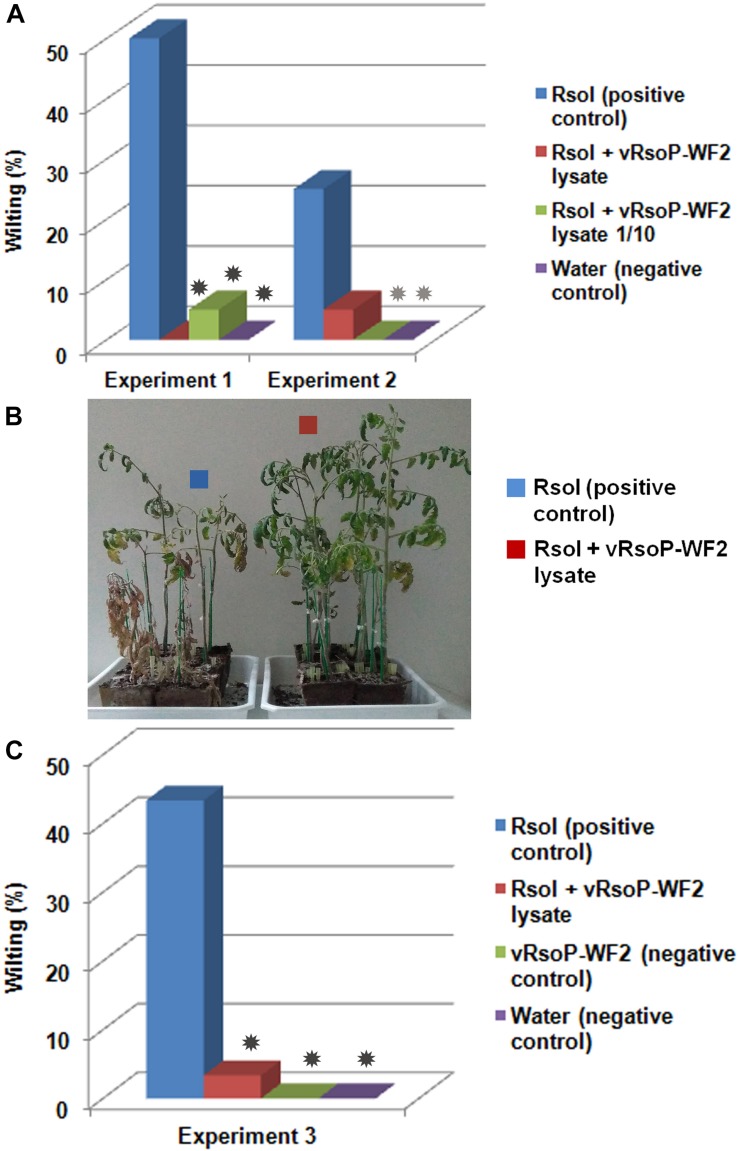
*Ralstonia solanacearum* biocontrol in host plants by watering with one single phage. **(A)** *In planta* biocontrol assays in tomato (*Solanum lycopersicum*) plants cv. Roma watered with 20 mL of *R. solanacearum* strain IVIA-1602.1 at 10^5^ colony-forming units (CFU)/mL (2 × 10^6^ CFU/pot) and the phage vRsoP-WF2 at 10^9^ or 10^6^ plaque-forming units (PFU)/mL or their 10-fold dilutions (from 2 × 10^10^ to 2 × 10^7^ PFU/pot) in irrigation water. Reductions in the incidence of bacterial wilt in the presence of vRsoP-WF2 were significant (*p* < 0.001) in Experiment 1 for both phage treatments, and also for the diluted lysate in Experiment 2 (*p* < 0.05). **(B)** Plants inoculated only with strain IVIA-1602.1 (positive controls) and biocontrol with the phage vRsoP-WF2 can be observed in the left and right side, respectively. Plants inoculated with water (negative controls) were negative. **(C)** *In planta* biocontrol assays in tomato (*S. lycopersicum*) plants cv. Roma watered with 20 mL of *R. solanacearum* strain IVIA-1602.1 at 10^5^ CFU/mL (2 × 10^6^ CFU/pot) and the phage vRsoP-WF2 at 10^8^ PFU/mL (2 × 10^9^ PFU/pot) in irrigation water. Reductions in the incidence of bacterial wilt in the presence of vRsoP-WF2 were significant (*p* < 0.001) in Experiment 3. Bars are the means of biocontrol assays carried out in duplicate (Experiments 1 and 2) or triplicate (Experiment 3) with 10 plants per condition. Asterisks indicate statistically significant differences with respect to the positive control (dark asterisk *p* ≤ 0.001; light asterisk *p* < 0.05).

Bacterial wilt biocontrol by the phage vRsoP-WF2 against the strain IVIA-1602.1 was observed in susceptible tomato plants cv. Roma after being watered once with a mixture of the phage and the pathogen in non-sterile environmental water. Assays performed by watering with 20 mL of *R. solanacearum* populations at 10^4^ CFU/mL (2 × 10^5^ CFU/pot) yielded no disease symptoms in all condition after 6–8 wpi, with or without phage. Biocontrol activity could be demonstrated in assays by watering the host with 20 mL of bacterial populations of about 10^5^ CFU/mL (2 × 10^6^ CFU/pot) mixed with the phage vRsoP-WF2 at 10^9^ or 10^6^ PFU/mL or their 10-fold dilutions (from 2 × 10^10^ to 2 × 10^7^ PFU/pot) in Experiments 1 and 2 (Figure 5A), and by watering the host with 20 mL of bacterial populations of 10^5^ CFU/mL (2 × 10^6^ CFU/pot) mixed with the phage vRsoP-WF2 at 10^8^ PFU/mL in Experiment 3 (Figure 5C). A representative picture of the results can be observed in Figure 5B. In the three independent experiments, percentage values of bacterial wilt incidence in the positive controls were 25–50%, while in tomato plants irrigated with the phage and the pathogen the values of disease incidence decreased to 0–5%. Plants inoculated only with phage vRsoP-WF2 or water showed no disease symptoms. Wilting percentages were significantly reduced (*p* < 0.001 or *p* < 0.05) in most of the phage treatments in Experiments 1 and 2 (Figure 5A) and (*p* < 0.001) in Experiment 3. *R. solanacearum*-like colonies were reisolated from wilted plants and PCR identified.

#### With One Single Phage and Their Combinations

**FIGURE 6:**
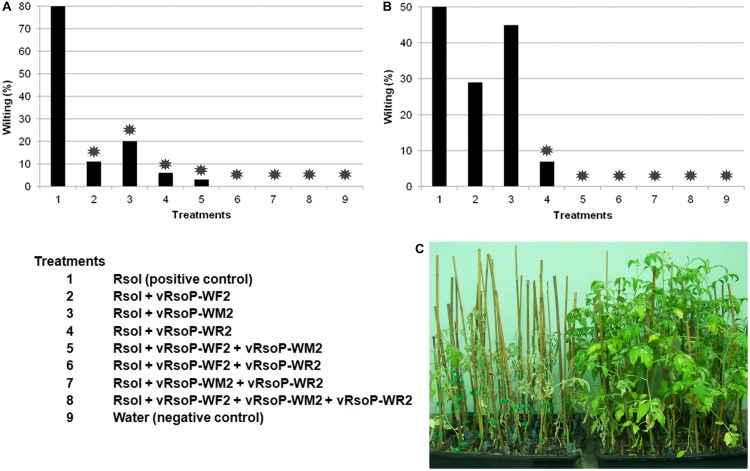
*Ralstonia solanacearum* biocontrol in host plants by watering with one single phage and their combinations. *In planta* biocontrol assays displaying reductions in the incidence of bacterial wilt in tomato (*Solanum lycopersicum*) plants cv. Roma watered with 20 mL of *R. solanacearum* strain IVIA-1602.1 at **(A)** 10^5^ colony-forming units (CFU)/mL (2 × 10^6^ CFU/pot) or **(B)** 10^6^ CFU/mL (2 × 10^7^ CFU/pot) and each of the three phages vRsoP-WF2, vRsoP-WM2, and vRsoP-WR2 separately or in mixtures of two and the three at 10^7^ plaque-forming units (PFU)/mL (2 × 10^8^ PFU/pot) in all experimental condition in irrigation water. Reductions in bacterial wilt percentages were significant (*p* < 0.001) in **(A)** all phage treatments applied, and **(B)** every mixture of two phages and the three, and also for phage vRsoP-WR2 when applied separately. **(C)** Plants inoculated only with strain IVIA-1602.1 (positive controls) and biocontrol with the mixture of the three phages can be observed in the left and right side, respectively. Plants inoculated with water (negative controls) were negative. Bars are the means of biocontrol assays performed with around 35 plants per condition. Asterisks indicate statistically significant differences with respect to the positive control (*p* < 0.001).

Bacterial wilt biocontrol assays were performed in susceptible tomato plants cv. Roma with each of the three phages vRsoP-WF2, vRsoP-WM2, and vRsoP-WR2 by watering them separately or in mixtures of two and the three against the strain IVIA-1602.1 in one single dose of 20-mL volumes of non-sterile environmental water, mimicking field conditions. Figure 6A shows results of a assay with initial concentrations of strain IVIA 1602.1 of about 10^6^ CFU/mL (2 × 10^7^ CFU/pot) and total initial concentration of phages of about 10^7^ PFU/mL (2 × 10^8^ PFU/pot). Bacterial wilt percentages obtained with positive controls were about 80% and values obtained after irrigation with single phages ranged between 20–6%, depending on the phage (Figure 6A). Decreases in bacterial wilt percentages up to 3–0% were observed when mixtures of binary combinations and all the three phages were used (Figure 6A). Reductions were significant (*p* < 0.001) in all the applied phage treatments (Figure 6A). Figure 6B shows results of a second assay with initial concentrations of strain IVIA 1602.1 of about 10^5^ CFU/mL (2 × 10^6^ CFU/pot) and total initial concentration of bacteriophages of about 10^7^ PFU/mL (2 × 10^8^ PFU/pot) in all experimental conditions. Percentages of bacterial wilt affected plants in the positive controls were about 50% and values obtained after irrigation with single phages ranged between 45–5%, depending on the phage (Figure 6B). Absence of bacterial wilt was observed when mixtures of binary combinations and the combination of the three phages were used (Figure 6B). Reductions in bacterial wilt percentages were significant (*p* < 0.001) in every mixture of two phages and the three, and also for phage vRsoP-WR2 when applied separately (Figure 6B). A representative picture of the results of the first experiment is shown in Figure 6C, where bacterial wilt positive controls and biocontrol with the mixture of the three phages can be observed in the left and right side, respectively. In both assays, negative control plants remained asymptomatic during the whole experimental period. Data indicated that mixtures of bacteriophages were more effective than single phages. The whole of non-wilted plants was kept under optimal disease development conditions for at least 2 months, and no late wilting was observed.

### vRsoP-WF2, vRsoP-WM2, and vRsoP-WR2 Are T7-Like Phages

Genomic DNA was obtained from each of the three bacteriophages, with A_260_/A_280_ ratios ranging from 1.92 to 2.02. After massive-scale sequencing, assembly of the obtained sequences yielded final assembled sequences with 100% fidelity. Genomic DNA sizes for the three bacteriophages were: 40,409 bp for SEQ ID N°1 (vRsoP-WF2), 40,861 bp for SEQ ID N°2 (vRsoP-WM2), and 40,408 bp for SEQ ID N°3 (vRsoP-WR2). Initial bioinformatic analysis and annotation pointed out that each of the major sequences in SEQ ID N°1 (vRsoP-WF2), SEQ ID N°2 (vRsoP-WM2), and SEQ ID N°3 (vRsoP-WR2) were easily identifiable as complete genomes of bacteriophages belonging to the genus of T7-like viruses, whose type species is the Enterobacteria phage T7, which belongs to the *Podoviridae* family.

Comparison of the genomes of the three bacteriophages showed >99% identity among them. Thus, between SEQ ID N°1 (vRsoP-WF2) and SEQ ID N°2 (vRsoP-WM2) as reference sequence, coverage and identity values were of 99 and 99.82%, respectively; between SEQ ID N°1 (vRsoP-WF2) and SEQ ID N°3 (vRsoP-WR2) as reference sequence, coverage and identity values were of 100 and 99.87%, respectively; and between SEQ ID N°2 (vRsoP-WM2) and SEQ ID N°3 (vRsoP-WR2) as reference sequence, coverage and identity values were of 98 and 99.77%, respectively. However, the analysis of these sequences revealed the presence of different small mutations, insertions and deletions distributed in their genomes. These differences were higher between SEQ ID N°2 (vRsoP-WM2) and the other two, SEQ ID N°1 (vRsoP-WF2) and SEQ ID N°3 (vRsoP-WR2). Thus, SEQ ID N°2 contains an insertion of 468 nucleotides in comparison to SEQ ID N°1 and SEQ ID N°3. The small differences found in the nucleotide sequences point out that vRsoP-WF2, vRsoP-WM2, and vRsoP-WR2 may be distinct bacteriophages of the same viral species. Bioinformatic analysis also indicated that they may be new phages, closely related to T7 bacteriophages. Genomes of vRsoP-WF2, vRsoP-WM2, and vRsoP-WR2 exhibited coverage and identity values of 97–98 and >99.7%, respectively, with *Ralstonia* phage RsoP1EGY (GenBank accession no. MG711516.1; Ahmad et al., 2018), and lower identity values, 98.8–98.9, 93.9–94.0, and 77.85%, with *Ralstonia* phage DU_RP_I (GenBank accession no. MF979559.1), *Ralstonia* phages P-PSG-11 and P-PSG-11-1 (GenBank accession no. MN270889.1 and MN270890.1), and *Ralstonia* phage RPSC1 (GenBank accession no. MF893341.1), respectively. Regions with high identity correspond to 5–23% of the complete genome of vRsoP-WF2, vRsoP-WM2, and vRsoP-WR2, and belong to highly conserved regions, mainly related to replication and encapsidation. Consequently, except in these conserved zones within the T7-like bacteriophages, the genomes of vRsoP-WF2, vRsoP-WM2, and vRsoP-WR2 seem to contain a nucleotide sequence divergent from that of the other bacteriophages deposited in GenBank. The three nucleotide sequences SEQ ID N°1 (vRsoP-WF2), SEQ ID N°2 (vRsoP-WM2), and SEQ ID N°3 (vRsoP-WR2) have been deposited in GenBank with accession numbers MN685189, MN685190, and MN685191, respectively.

## Discussion

Key to any successful application of phages against bacterial crop diseases is their discovery and subsequent characterization (Abedon et al., 2017; Buttimer et al., 2017; Svircev et al., 2018) as well as their persistence in natural settings (Balogh et al., 2010). In this work, three lytic bacteriophages (vRsoP-WF2, vRsoP-WM2, and vRsoP-WR2) were selected from a collection isolated by enrichment of *R. solanacearum*-contaminated environmental water samples from geographically distant origins in Spain, confirming the choice of environmental reservoirs as the better sources for antibacterial phages (Mattila et al., 2015). Among numerous approaches to characterize phage effectiveness (Weber-Dąbrowska et al., 2016), the range of targeted bacteria is one of the most important (Mirzaei and Nilsson, 2015), since a phage should display reasonable specificity (Abedon et al., 2017), especially for being used in the open field. The three bacteriophages proved to specifically infect a collection of phytopathogenic *R. solanacearum* (former phylotype II) strains, known to be a genetically homogeneous group (Fegan and Prior, 2005; Cellier and Prior, 2010; Safni et al., 2014), whereas non-target pathogenic or environmental bacteria were not affected, thus avoiding an impact on either host bacterial endophytes (Svircev et al., 2018) or other bacteria from the surroundings, as required for a safe biocontrol agent (Abedon et al., 2017; Buttimer et al., 2017). The phages vRsoP-WF2, vRsoP-WM2, and vRsoP-WR2 constitute up until now the first isolated from environmental water with lytic activity and specificity against the present species *R. solanacearum*. Only recently, other two lytic phages against *R. solanacearum* were reported, both from soil (Ahmad et al., 2018; Elhalag et al., 2018; Addy et al., 2019). However, other phages, whether isolated from soil or wilted hosts, were described to be active against *R. pseudosolanacearum* and/or *R. syzygii* subsp. *indonesiensis* but, not against *R. solanacearum*, as reviewed (Álvarez and Biosca, 2017).

The three phages also displayed effective lytic activity on *R. solanacearum* populations within a wide range of environmental conditions established in freshwater from river, lake, and irrigation sources with different values of water temperature, pH, salinity contents, and also lack of aeration mimicking water storage tanks, all of them factors influencing the infection cycle in the natural settings (Ly-Chatain, 2014). Other phages reported were active against *R. solanacearum*, *R. pseudosolanacearum*, or *R. syzygii* subsp. *indonesiensis* at a range of temperature and/or pH values but, none of them was assayed in any kind of environmental water (Fujiwara et al., 2011; Bae et al., 2012; Lee and Park, 2016; Álvarez and Biosca, 2017; Elhalag et al., 2018; Addy et al., 2019), which is a requirement for phage application through irrigation water. It is remarkable to mention that, in this work, assays to establish the range of environmental conditions for phage activity were carried out in cocultures of *R. solanacearum*:phage, and so lysis was always determined by the range of *R. solanacearum* physiological conditions. The phages vRsoP-WF2, vRsoP-WM2, and vRsoP-WR2 proved to be lytic under a variety of realistic conditions and a host range allowing productive infection on the targeted pathogen, displaying qualities suitable for biocontrol application, as indicated (Buttimer et al., 2017).

Morphological traits of the three phages were characteristic of the *Caudovirales*, *Podoviridae* family, as other reported tailed phages affecting *R. solanacearum*, *R. pseudosolanacearum*, or *R. syzygii* subsp. *indonesiensis* (Kawasaki et al., 2009; Fujiwara et al., 2011; Bae et al., 2012; Bhunchoth et al., 2015; Elhalag et al., 2018; Liao, 2018). The use of podoviruses as biocontrol agents has been described with variable results to fight either bacterial wilt caused by *R. solanacearum* (Elhalag et al., 2018), *R. pseudosolanacearum*, and/or *R. syzygii* subsp. *indonesiensis* (Fujiwara et al., 2011; Bae et al., 2012; Bhunchoth et al., 2015), or other plant diseases (Buttimer et al., 2017).

The phages vRsoP-WF2, vRsoP-WM2, and vRsoP-WR2 showed high efficiency of infection of *R. solanacearum*, with reductions in the bacterial populations of several orders of magnitude in just a few hours. This was similarly observed with phages active against *R. pseudosolanacearum* and/or *R. syzygii* subsp. *indonesiensis*, as ϕRSA1 and ϕRSB1, whereas the jumbo ϕRSL1 had an infection cycle rather longer than the doubling time of host cells, requiring a higher dose to efficiently infect (Fujiwara et al., 2011). Therefore, phages should be able to quickly lyze the host, multiply and disseminate in the environment to which they were applied (Buttimer et al., 2017). Effective lysis of *R. solanacearum* by the three phages was confirmed at different initial bacterium:phage concentrations and at several temperatures. Reductions in the *R. solanacearum* populations were delayed in time because of either lower initial phage concentration or lower temperature, the latter confirming slower biotic interactions as stated (Álvarez et al., 2007), probably due to lower metabolic activity of the host. Likewise, lytic activity of podovirus RsPod1EGY against *R. solanacearum* was detected at different MOIs (Elhalag et al., 2018), and the growth of myovirus RsoM1USA-treated *R. solanacearum* was significantly reduced in comparison to the untreated bacterial cells (Addy et al., 2019). However, phages with similar lytic activity against *R. solanacearum*, even belonging to the same virus family, may have different potential biocontrol *in planta* (Addy et al., 2019). Despite the fact that infection of *R. pseudosolanacearum* and/or *R. syzygii* subsp. *indonesiensis* by the jumbo myovirus ϕRSL1 took longer than that by myovirus ϕRSA1 and podovirus ϕRSB1, treatment with ϕRSL1 resulted in better plant-protecting effects (Fujiwara et al., 2011).

Extended survival of lytic phages in the natural settings keeping their capability to suppress bacterial populations is certainly a desirable quality for a successful plant disease biocontrol agent (Balogh et al., 2010). The phages vRsoP-WF2, vRsoP-WM2, and vRsoP-WR2 maintained effective lytic activity against *R. solanacearum* in environmental water for more than 5 months at 4, 14, and 24°C and up to 3 years at 4 and 14°C. This is the longest persistence reported until now for phages with biocontrol potential, showing the high stability of the three phages in environmental water, in the absence of the host, and suggesting stability in field application. No data are available at this respect about other *R. solanacearum*-infecting bacteriophages. Concerning phages known to be active against *R. pseudosolanacearum* and/or *R. syzygii* subsp. *indonesiensis*, jumbo myovirus ϕRSL1 was retained in soil and roots of host plants for four months, whereas survival of podovirus PE204 in soil was found to be temperature dependent, keeping stable at 25 and 30°C over 13 days but, losing infectivity at 35°C after 10 days; in both cases, the presence of *R. solanacearum* comparatively enhanced phage stability (Fujiwara et al., 2011; Bae et al., 2012).

Effective biocontrol activity against *R. solanacearum* populations in environmental water was consistently demonstrated by one representative of the three phages, vRsoP-WF2, with strong reductions in bacterial cells of about 5 log units within 10 h at 24°C and 3 log units within 23 h at 14°C, at an initial MOI of 0.01. No available data can be found at this respect about other *R. solanacearum*-infecting phages, since their biocontrol efficacy was tested only *in planta* (Elhalag et al., 2018; Addy et al., 2019), and in soil or *in planta* for phages with activity against *R. pseudosolanacearum* and/or *R. syzygii* subsp. *indonesiensis* (Álvarez and Biosca, 2017). Appearance of phage-resistant *R. solanacearum* cells took place at both temperatures but, only in conditions of bacterium:phage cocultures, with both types, wild and resistant, growing simultaneously on plates at 14°C. Development of bacterial resistance to phages is considered one of the main barriers that may hamper the effectiveness of a bacteriophage-based biocontrol method (Ly-Chatain, 2014; Buttimer et al., 2017; Svircev et al., 2018). With respect to phages active against *R. pseudosolanacearum* and/or *R. syzygii* subsp. *indonesiensis*, virulent myovirus ϕRSA1 and podovirus ϕRSB1 were discarded for biocontrol use because they failed to stably prevent bacterial growth, since resistant cells were observed in the *in vitro* and *in planta* experiments, even in a cocktail together with jumbo myovirus ϕRSL1 (Fujiwara et al., 2011). Interestingly, in this study, durable long-term biocontrol was observed in non-sterile environmental water with the whole indigenous microbiota, where no resistant *R. solanacearum* cells were observed, probably because emerging resistant bacterial populations would find it difficult to thrive in an established ecosystem. Okabe and Goto (1963) also reported the impossibility to reproduce *in planta* the appearance of resistant bacteria that they had observed *in vitro* and native water microbiota was described to have a deleterious effect on *R. solanacearum* populations (van Elsas et al., 2001; Álvarez et al., 2007). Currently, there are no other reported methods available to effectively control this pathogen in contaminated waterways despite water is an increasingly scarce resource and many *R. solanacearum* susceptible crops are irrigated.

Moreover, the phages vRsoP-WF2, vRsoP-WM2, and vRsoP-WR2 successfully proved to be effective for bacterial wilt biocontrol *in planta* by watering with either one phage or mixtures of two or the three phages against *R. solanacearum* in non-sterile environmental conditions, mimicking those of the natural settings, and suggesting that phage application through irrigation can also be considered for soils. Significant decreases in bacterial wilt percentages were observed after irrigation of tomato plants with single phages in the course of two bacterial wilt biocontrol assays performed at MOIs of 100 and 10, and stronger reductions or even absence of bacterial wilt incidence were achieved when mixtures of binary combinations and/or the combination of the three phages were used, pointing out that mixtures were more effective than single phages, as generally stated (Ly-Chatain, 2014; Buttimer et al., 2017; Svircev et al., 2018) with just few exceptions (Fujiwara et al., 2011). No late wilting in asymptomatic plants was observed, so discarding the presence of *R. solanacearum* resistant cells in the non-sterile environmental conditions, similarly to Okabe and Goto (1963) and Álvarez et al. (2007).

Biocontrol potential by other *R. solanacearum*-infecting phages was also reported. Soilborne podovirus RsPod1EGY seemed to be effective in suppression of wilting symptoms during 10 days under greenhouse conditions in assays with 12 tomato plants pretreated with 10^12^ PFU/pot but, cutting the roots to ease an artificial inoculation (Elhalag et al., 2018). On the other hand, soilborne myovirus RsoM1USA did not reduce *R. solanacearum* virulence in tomato plants (Addy et al., 2019). This phage is closely related to myovirus ϕRSA1, which also proved to be unsuccessful for bacterial wilt biocontrol (Fujiwara et al., 2011; Addy et al., 2019). From all the phages reported active against *R. pseudosolanacearum* and/or *R. syzygii* subsp. *indonesiensis in vitro*, only two of them demonstrated biocontrol potential *in planta*, the jumbo myovirus ϕRSL1 and the podovirus PE204 (Fujiwara et al., 2011; Bae et al., 2012; Álvarez and Biosca, 2017). Surprisingly, phage PE204 is highly similar to podovirus ϕRSB1, which failed to control bacterial wilt *in planta* (Fujiwara et al., 2011; Bae et al., 2012), and confirming the fact that suitable *in vitro* potential of phages does not necessarily correspond to successful biocontrol potential *in planta*, making it necessary to carry out a detailed biological characterization of each of them (Buttimer et al., 2017; Addy et al., 2019).

As vRsoP-WF2, vRsoP-WM2, and vRsoP-WR2 proved to gather appropriate qualities for bacterial wilt biocontrol, they were subsequently identified, and further genetic characterization is underway. They had genome sizes around 40 kbp, which fits the range of podoviruses (Lavigne et al., 2003). Similarly, podovirus RsPod1EGY active against *R. solanacearum* had 41,297 bp of DNA (Ahmad et al., 2018), and genome of podoviruses ϕRSB1 and RPSC1 against *R. pseudosolanacearum* and/or *R. syzygii* subsp. *indonesiensis* comprised 43,079 bp (Kawasaki et al., 2009) and 39,628 bp (Liao, 2018), respectively. Although there was high identity among the genomes of the three phages, mutations, insertions, and deletions suggested that vRsoP-WF2, vRsoP-WM2, and vRsoP-WR2 may be new, distinct members of the *T7likevirus* genus, type species Enterobacteria phage T7, *Podoviridae* family. The three seem closely related to the phage RsoP1EGY (Ahmad et al., 2018), since identity was higher than 99.7%, although with 97–98% coverage and different genome sizes (436–889 nucleotides longer the phage RsoP1EGY). T7-like phages are generally known as obligately lytic phages, which is convenient for the application of a phage as a biocontrol agent. Also a short lytic cycle, characteristic of T7-like phages, is considered a desirable attribute for a biocontrol agent (Hamdi et al., 2016). Among podoviruses active against *R. pseudosolanacearum* and/or *R. syzygii* subsp. *indonesiensis*, the phage ϕRSB1 was assigned as T7-like (Kawasaki et al., 2009) but, unsuccessfully tested for bacterial wilt biocontrol (Fujiwara et al., 2011). However, T7-like phages are particularly interesting for biocontrol applications, since their small genomes would be hindering the transfer of DNA fragments with pathogenicity islands to the host (Krylov, 2001). T7-like phages also have a conserved organization, favoring their in-depth analysis (Zhu et al., 2010). Genomes of vRsoP-WF2, vRsoP-WM2, and vRsoP-WR2 shared a similar genomic organization to coliphage T7 and T7-like viruses, and contained conserved replication and encapsidation regions with high identity to those of other T7-like phages. More detailed genetic characterization of the genome of the three phages is presently being carried out. Genome organization and sequence similarity to existing phages strongly support the assignment of the three phages active against *R. solanacearum* as members of the *T7likevirus* genus.

## Conclusion and Perspectives

Effective and environmentally friendly biocontrol of *R. solanacearum* populations by three new lytic phages presented in this work, vRsoP-WF2, vRsoP-WM2, and vRsoP-WR2, was observed for all of them and their combinations in irrigation water and host plants, with high reductions in bacterial wilt incidence. These new phages were isolated from environmental water and proved to be specific against *R. solanacearum* and some strains of *R. pseudosolanacearum*. Their remarkable long-term persistence in environmental water while keeping their lytic activity makes it feasible their successful application in irrigation water as well as in host plants by watering. Such ability as biocontrol agents may allow their use in the field or greenhouse conditions and constitutes a novel ecological strategy for these phages to be combined and easily applied through irrigation water for bacterial wilt prevention and control of tomato and other irrigated crops in the field. This innovative bacteriophage-based method focused on: (i) the three new phages being the first isolated from environmental water with activity against *R. solanacearum*, (ii) displaying the longest persistence in environmental water reported until now for phages with biocontrol potential, and (iii) consistently proving to be able to control the disease in the host plant under environmental conditions. It has been the subject of a recent patent in order to be effectively used with other biocontrol strategies in an integrated management program with less impact than agrochemicals and closer approach to sustainability of agricultural systems.

## Data Availability Statement

All datasets generated for this study are included in the article/supplementary material.

## Author Contributions

BÁ, ML, and EB planned and designed the experiments and wrote the manuscript. BÁ performed the laboratory assays. BÁ and EB carried out the assays with host plants under BSL3 conditions.

## Conflict of Interest

The University of Valencia (UV) and the Valencian Institute for Agricultural Research (IVIA) have the following interests: a granted Spanish patent ES2592352 B2 and the patents filed in EU and EEUU–P16799402.9 and US15/576,798.
